# Innately Fluorescent Tetravalent Cytotoxic Conjugate TetraF_HER2_-vcMMAE Engages Aggregation-Dependent Endocytosis of HER2 for Enhanced Intracellular Drug Delivery

**DOI:** 10.1021/acs.jmedchem.5c00782

**Published:** 2025-06-29

**Authors:** Natalia Porębska, Aleksandra Chorążewska, Krzysztof Ciura, Adam Pomorski, Artur Krężel, Łukasz Opaliński

**Affiliations:** † Department of Medical Biotechnology, Faculty of Biotechnology, 49572 University of Wroclaw, F. Joliot-Curie 14a, 50-383 Wroclaw, Poland; ‡ Department of Chemical Biology, Faculty of Biotechnology, University of Wroclaw, F. Joliot-Curie 14a, 50-383 Wroclaw, Poland

## Abstract

Breast cancer is the most common malignancy in women, with approximately 20–30% of all diagnosed cases characterized by HER2 overexpression. Several HER2-targeted cytotoxic conjugates have been developed, but their efficacy is limited. One of the main obstacles restraining the effectiveness of HER2-specific cytotoxic conjugates is their low internalization, as HER2 is immobile mainly on the cell surface. Therefore, there is a need to develop novel HER2-selective cytotoxic conjugates that will overcome HER2 immovability and, by this, ensure efficient drug delivery into HER2-overexpressing cancer cells. Here, we present a novel system for generating high affinity, self-assembling, inherently fluorescent, and multivalent HER2 ligands. The developed HER2-specific ligands largely overcome the innate stability of HER2 in the plasma membrane by triggering clathrin-independent aggregation-dependent endocytosis of the receptor. To exploit the pro-endocytic potential of developed proteins, we constructed the tetravalent fluorescent cytotoxic conjugate TetraF_HER2_-vcMMAE and demonstrated its high potency and selectivity against HER2+ breast cancer cells.

## Graphical abstract

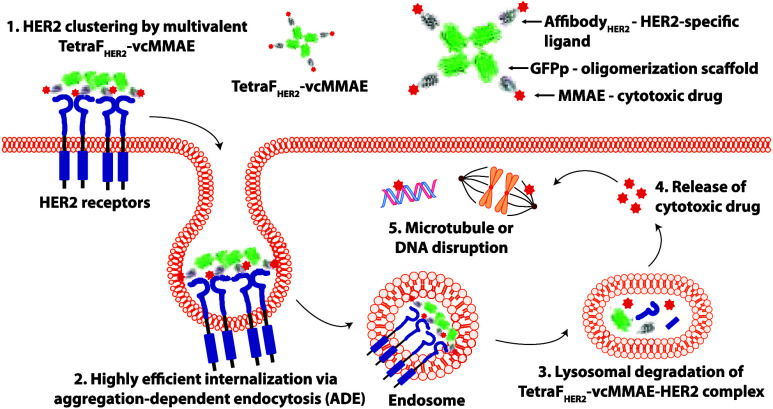

## 1 Introduction

Breast cancer is the most common cancer type among women that globally caused over 670 000 deaths in 2022.^1^ Based on histological and molecular characteristics, breast tumors are divided into four major groups: luminal A, luminal B, human epidermal growth factor receptor 2-positive (HER2+) and triple-negative breast cancer (TNBC).^2,3^ HER2+ is an aggressive subtype of breast cancer and is associated with a worse prognosis for patients. The HER2 receptor is overexpressed in 20–30% of breast tumors and is considered to be one of the major oncogenic drivers in breast cancer.^4,5^ HER2 is a member of the epidermal growth factor receptor family (EGFR) of receptor tyrosine kinases (RTKs).^4,6^ HER2 is 185 kDa multidomain glycosylated cell surface receptor involved in signaling pathways like mitogen-activated protein kinase (MAPK), phosphatidylinositol 3-kinase (PI3K), protein kinase B (AKT), and the mammalian target of rapamycin (mTOR).^4,7,8^ HER2-dependent signaling regulates pivotal cellular processes, like cell division, motility, differentiation and play a fundamental role in the physiological growth and differentiation of breast tissue.^4,8,9^ HER2 is considered an orphan receptor, because no HER2 ligands have been identified so far. Therefore, the oncogenic mechanism of HER2 is ligand-independent and relies mainly on the ability of HER2 to form heterodimers with other RTKs, leading to their activation and the propagation of signals that sustain cancer cell survival, proliferation, and migration.^8–10^ The oncogenic activity of HER2 is not limited to breast cancer. HER2 is also overexpressed in several other tumors, like gastric cancer, nonsmall cell lung cancer, biliary tract cancer, bladder cancer or colorectal cancer.^11–14^ Several HER2-targeted therapeutic approaches have been developed, including HER2 tyrosine kinase inhibitors, monoclonal antibodies, and antibody–drug conjugates (ADCs).^3,7,11,15^ ADCs are modern medicines that act as precision-guided “biological missiles”, effectively delivering cytotoxic drugs to cancer cells while avoiding healthy cells.^16,17^ ADCs comprise a monoclonal antibody coupled to a highly cytotoxic drug *via* a specific linker. The monoclonal antibody in the ADC recognizes the target receptor overproduced by cancer cells and ensures cellular uptake of the ADC *via* receptor-mediated endocytosis. Inside the cancer cell, ADC is transported through endosomal compartments to the lysosomes, where the antibody and linker proteolysis occur, releasing the active drug form. Due to its hydrophobicity, the free drug crosses the lysosomal membrane and reaches its final cellular target (*e.g.*, tubulin or topoisomerase), leading to apoptosis of the cancer cells.^18–20^ In addition to antibodies and their fragments, other macromolecules, such as receptor ligands and peptides that specifically recognize cancer-overproduced receptors, are used as drug delivery agents in cytotoxic conjugates. Two HER2-specific ADCs, trastuzumab emtansine (T-DM1) and trastuzumab deruxtecan (T-DXd), have been approved for the treatment of metastatic breast cancer and proven effective for some patients who have previously received the anti-HER2 monoclonal antibody (trastuzumab) and chemotherapy.^15,21,22^ The major drawback of T-DM1 and T-DXd, in addition to partially limited effectiveness, is the high therapy cost. In addition, metastatic HER2+ tumors inevitably develop resistance through various mechanisms, leading to disease progression.^21,23,24^ Therefore, the development of more potent therapeutic approaches for targeting HER2-overexperssing cancer cells is urgently needed to increase the availability of treatment, extend patients’ lives, and improve their quality of life.

A critical step for the efficiency and specificity of conjugates targeting HER2 is their rapid and selective internalization into cancer cells *via* receptor-mediated endocytosis.^25^ However, HER2 is considered a low-internalizing receptor, which, even when endocytosed, is frequently recycled to the plasma membrane, avoiding lysosomal degradation.^25–27^ The exact mechanisms of this phenomenon are not fully understood. It has been suggested that the stabilization of HER2 on the cell surface occurs through the interaction of HER2 with other proteins, such as Hsp90 or flotillins.^28–31^ Novel strategies to improve endocytosis and lysosomal trafficking of HER2 are awaited to improve the effectiveness of targeted therapies against HER2-overproducing cancers. Recent findings of Paul et al. and of our group demonstrate that the clustering of selected receptors on the cell surface, including HER2, triggered by extracellular multivalent ligands essentially enhances the efficiency of receptor endocytosis, implicating the presence of inherent endocytic pathway ensuring rapid removal of cell surface aggregates.^32–34^ Here, we decided to develop novel multivalent HER2 ligands capable of HER2 clustering and overcoming HER2 immobility and to use selected proteins as highly effective cytotoxic drug carriers in the protein drug conjugate (PDC) strategy targeting HER2+ breast cancer cells.

## 2 Results

### 2.1 Engineering of Intrinsically Fluorescent, Multivalent HER2-Specific Ligands

In order to develop multivalent, inherently fluorescent HER2-specific ligands, we decided to fuse Affibody_HER2:342_ (HER2 ligand) with green fluorescent protein polygons GFPp (oligomerization scaffold) (Figure 1A). Affibody_HER2:342_ is a three-helix protein derived from *Staphylococcal* protein A capable of binding the extracellular domain of HER2 with high selectively and high affinity (Figure 1A).^35^ As a scaffold for controlled oligomerization of Affibody_HER2:342_, we employed GFPp, which was developed by Kim et al. GFPp are GFP variants in which a single β-strand was transferred to the other part of the β-barrel.^36^ This prevents folding of the fluorogenic β-barrel of GFPp but facilitates intermolecular GFPp interactions, leading to the GFPp oligomerization and assembly of the fluorogenic GFPp β-barrel in oligomers due to the complementation of the missing β-strand (Figure 1A). Designed GFPp-Affibody_HER2:342_ fusion protein should result in self-assembling, intrinsically fluorescent, multivalent, and HER2-specific ligands. GFPp_Affibody_HER2:342_ was successfully expressed in the bacterial protein expression system and purified with affinity chromatography (Figure S1A). The purity and the identity of the GFPp_Affibody_HER2:342_ were confirmed by SDS-PAGE (Figure 1B), Western blotting (Figure 1C), and mass spectrometry (Figure 1D).

**Figure 1:**
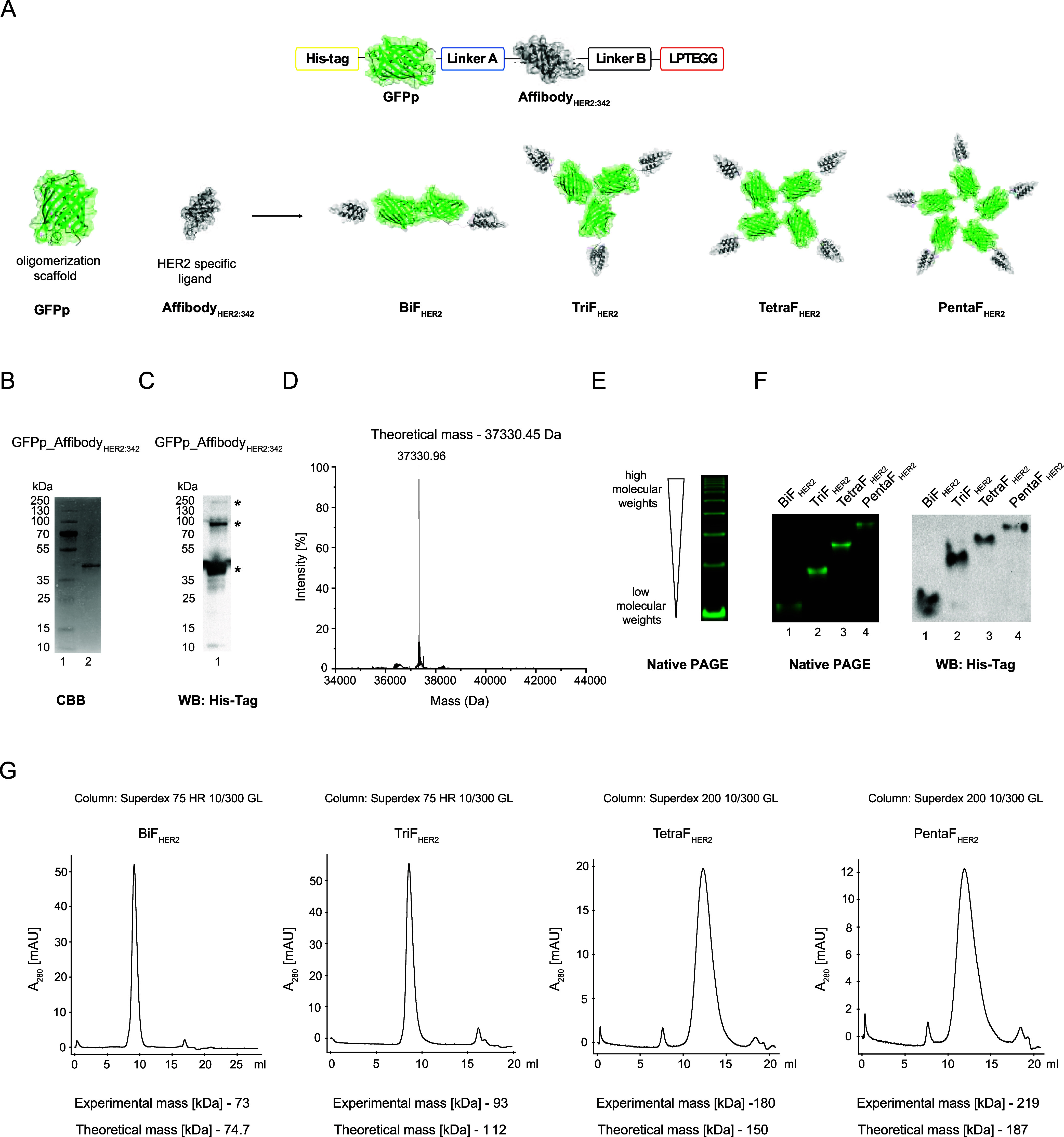
Engineering of fluorescent, multivalent HER2-specific ligands. (A) Schematic representation of the engineered fluorescent multivalent GFPp_Affibody_HER2:342_ oligomers. The mixture of GFPp_Affibody_HER2:342_ oligomers was purified by affinity chromatography and analyzed using SDS-PAGE (B) and Western blotting with anti-His-Tag antibodies. Asterisks mark nonfully denatured higher oligomeric forms of multivalent HER2 ligands in (C). (D) The identity of GFPp_Affibody_HER2:342_ was confirmed by mass spectrometry. (E) The mixture of GFPp_Affibody_HER2:342_ oligomers was separated under nondenaturing conditions by Native PAGE. The fluorescence properties of the purified oligomers were assessed by UV imaging of Native PAGE gels. (F) The efficacy of isolating different oligomeric forms was confirmed by Native PAGE and Western blotting with anti-His-Tag antibodies. (G) The oligomeric state of the purified proteins was assessed by size exclusion chromatography (SEC). The slight differences in the masses are because, in this technique, the separation of the oligomers is also influenced by their shape.

Next, we used the intrinsic fluorescence of GFPp_Affibody_HER2:342_ in conjunction with native PAGE in order to study the oligomeric states of developed HER2 ligands. As shown in Figure 1E, purified GFPp_Affibody_HER2:342_ was identified in multiple fluorogenic bands, implicating successful Affibody_HER2:342_ oligomerization by GFPp. After GFPp_Affibody_HER2:342_ separation by native PAGE, we cut out individual bands representing particular oligomers and eluted proteins from the gel. Using this approach, we obtained highly pure, fluorescent HER2-specific multivalent variants of GFPp_Affibody_HER2:342_: the dimeric bivalent (BiF_HER2_), trimeric trivalent (TriF_HER2_), tetrameric tetravalent (TetraF_HER2_) and pentameric pentavalent (PentaF_HER2_), as demonstrated with native PAGE followed by ultraviolet (UV) detection (Figure 1F) and Western blotting (Figure 1G). The assembly of specific oligomeric states of isolated GFPp_Affibody_HER2:342_ variants was also confirmed by calibrated size exclusion chromatography (SEC) (Figure 1G). These data implicate the successful design and isolation of HER2-specific ligands of distinct valency.

### 2.2 Stability of GFPp-Affibody_HER2:342_ Oligomers

To serve as potential drug carriers, developed proteins should display high stability. Therefore, we assessed the stability of GFPp_Affibody_HER2:342_ oligomers by incubating individual variants in a serum-free medium and analyzing their total levels (SDS-PAGE) and the oligomeric state (native PAGE) in time. As shown in Figure 2A, the total level and the oligomeric state (Figure 2B) of all studied HER2-specific ligands have not changed even after 96 h of incubation at 37 °C. In addition, we measured the stability of the GFPp oligomerization scaffold by monitoring GFP fluorescence after incubation of the oligomers in 10-fold diluted human serum. As shown in Figure 2C, the fluorescence of all developed HER2 ligands remained unchanged even after 96 h incubation in serum. These data demonstrate that the oligomeric state and fluorogenic properties of developed HER2 ligands remain virtually intact for at least 96 h in the serum, suggesting the high stability of all developed HER2-specific fluorogenic ligands.

**Figure 2:**
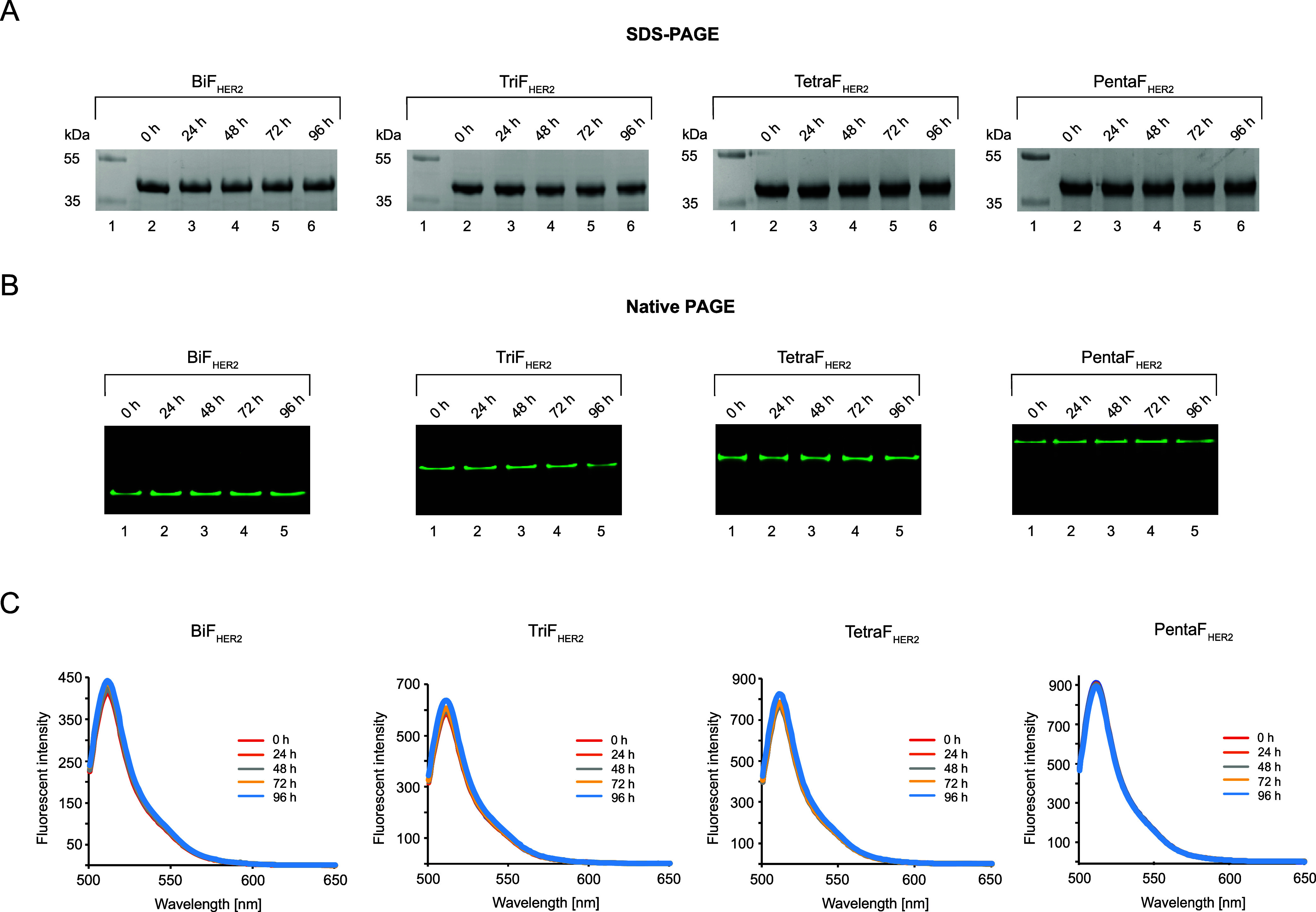
Stability analysis of multivalent HER2-specific ligands. The oligomers were incubated in a serum-free medium at 37 °C for 96 h. At distinct time points, samples were taken, and the stability of the GFPp_Affibody_HER2:342_ oligomers was analyzed by SDS-PAGE (A) and UV imaging of Native PAGE gels (B). (C) The stability of the GFPp oligomerization scaffold was confirmed by monitoring GFP fluorescence emission at different points of incubation of the oligomers in 10-fold diluted human serum at 37 °C. Fluorescence spectra were acquired with excitation at 488 nm and emission in the 500–650 nm range. Representative data from three independent experiments are shown.

### 2.3 Superior Affinity of Multivalent Ligands for HER2

We used confocal microscopy to assess the specificity of the generated multivalent ligands for HER2. To this end, we used HER2+ BC cells, SKBR-3, and the HER2- BC model, MCF-7. Initially, we confirmed HER2 expression in these cell lines using Western blotting (Figure 3A). We incubated SKBR-3 and MCF-7 cells cold (to prevent endocytosis and visualize cell binding) with BiF_HER2_, TriF_HER2_, TetraF_HER2_ and PentaF_HER2_, extensively washed cells, and employed intrinsic fluorescence of multivalent ligands to determine their specificity for HER2. As shown in Figure 3B, all studied ligands displayed strong fluorescent signals on the surface of the SKBR-3 cells, and we detected no staining of MCF-7. These data demonstrate that developed ligands are highly specific for HER2 and can recognize HER2 exposed on the cell surface. To determine whether the oligomeric proteins can direct HER2 binding, we performed qualitative binding tests using native PAGE and purified proteins. The particular oligomers were incubated with the recombinant extracellular domain of HER2 (HER2.ecd-Fc), and the ligand–receptor complexes were visualized with native PAGE by detecting the fluorescence of GFPp and by Western blotting using anti-HER2 antibodies, confirming the presence of HER2 in a high molecular weight complex with HER2-specific ligands. As shown in Figure 3C, in all cases, the addition of HER2.ecd-Fc to BiF_HER2_, TriF_HER2_, TetraF_HER2_, and PentaF_HER2_ promoted the appearance of slowly migrating fluorescent bands (absent in HER2.ecd-Fc—non treated controls) that also contained HER2, as judged from Western blotting with anti-HER2 antibodies. These data indicate that all HER2-specific fluorogenic ligands directly interact with the extracellular domain of HER2.

**Figure 3:**
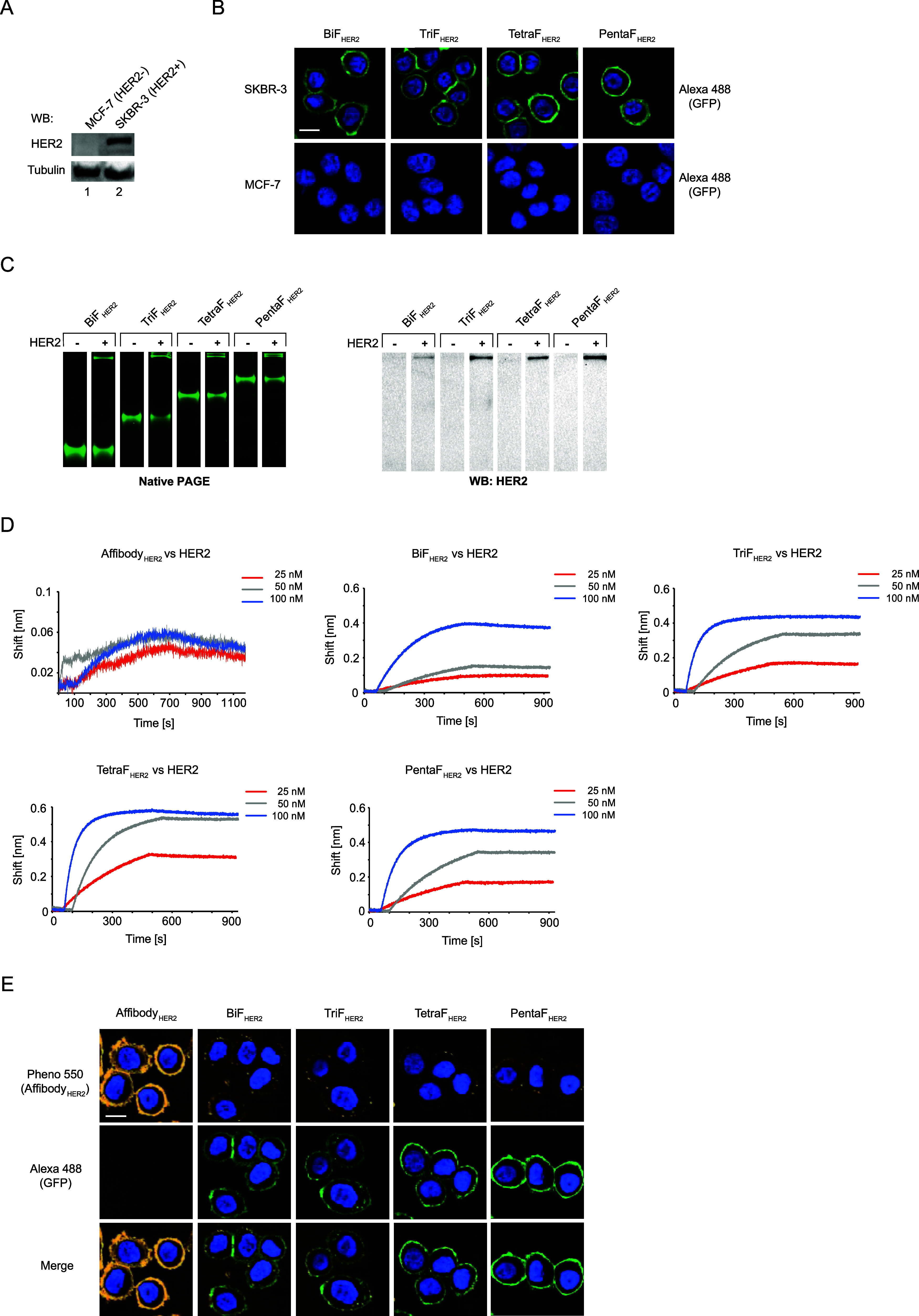
Analysis of the affinity of multivalent ligands for HER2. (A) The expression level of HER2 in SKBR-3 and MCF-7 cell lines was analyzed by Western blotting. Tubulin levels were used as loading control. (B) The specificity of the interaction between GFPp_Affibody_HER2:342_ oligomers and HER2 receptor was confirmed by confocal microscopy. SKBR-3 and MCF-7 cells were incubated with 300 nM BiF_HER2_, TriF_HER2_, TetraF_HER2_, and PentaF_HER2_ for 30 min on ice. Nuclei were stained with NucBlue Live dye, and cells were fixed in a 4% paraformaldehyde solution. (C) GFPp_Affibody_HER2:342_ oligomers were incubated with the recombinant extracellular domain of HER2 (HER2.ecd-Fc) for 15 min at room temperature, and the formation of the ligand–receptor complex was confirmed by Native PAGE and Western blotting using anti-HER2 antibodies. Oligomers without added receptors were used as a control. (D) BLI analyses of the interaction between GFPp_Affibody_HER2:342_ oligomers and the HER2 receptor. HER2-Fc was immobilized on Protein A sensors, and the association and dissociation phases were monitored at different protein concentrations. A reference sensor without HER2-Fc was used as a control. (E) Microscopic analysis of the enhanced binding of multivalent variants to HER2. SKBR-3 cells were preincubated with DyLight 550-labeled monomeric Affibody_HER2:342_ for 10 min on ice, then equimolar concentrations of BiF_HER2_, TriF_HER2_, TetraF_HER2_, or PentaF_HER2_ were added, and incubation was continued for 30 min. Cells incubated with monomeric proteins were used as a control. Nuclei were stained with NucBlue Live dye, and the cells were fixed in a 4% paraformaldehyde solution. Representative images from three independent experiments are shown. The scale bar is 20 μm.

An effective targeting molecule should display a high affinity for the receptor to allow for its precise recognition and preferably also the kinetics of binding that supports the formation of a stable complex on the cell surface that will last long enough to facilitate the assembly of the endocytic machinery. To quantitatively assess the impact of increasing the valency of developed ligands on the interaction with HER2, we produced a control, monomeric Affibody_HER2:342_ (Figure S1B). The extracellular region of HER2 was immobilized on Protein A sensors and incubated with BiF_HER2_, TriF_HER2_, TetraF_HER2_, and PentaF_HER2_ or with the monomeric Affibody_HER2:342_ as a control, and the parameters of the interaction were measured with BLI. As expected, all studied HER2 ligands directly interacted with the HER2 receptor, which confirms previous results (Figure 3D). Furthermore, all developed fluorescent multivalent HER2 ligands displayed higher affinity for HER2 as compared with the monomeric Affibody_HER2:342_ (Figure 3D and Table 1). The kinetic parameters showed enhanced association rates and primarily decreased dissociation rates of multivalent ligands, especially for variants of higher valency: TriF_HER2_, TetraF_HER2_ and PentaF_HER2_ in relation to the monomeric Affibody_HER2:342_ (Figure 3D and Table 1). These data indicate that multivalency improved the binding of Affibody_HER2:342_ to HER2. Oligomeric HER2 ligands bind HER2 with nanomolar affinity and form long-lasting complexes with the receptor.

**Table 1:** Kinetic Parameters of the Interaction between GFPp_Affibody_HER2:342_ Proteins and HER2^a^

HER2-Fc	*K*_D1_ [M]	*K*_D2_ [M]	*K*_on1_ [1/Ms]	*K*_on2_ [1/Ms]	*K*_off1_ [1/s]	*K*_off2_ [1/s]
Affibody_HER2:342_	7.71 × 10^–08^	7.09 × 10^–07^	6.46 × 10^04^	1.92 × 10^04^	1.32 × 10^–03^	1.54 × 10^–02^
BiF_HER2_	2.57 × 10^–12^	1.32 × 10^–08^	9.98 × 10^04^	9.46 × 10^04^	2.23 × 10^–07^	1.05 × 10^–03^
TriF_HER2_	1.47 × 10^–12^	2.74 × 10^–09^	1.03 × 10^05^	1.44 × 10^05^	1.55 × 10^–07^	3.50 × 10^–04^
TetraF_HER2_	1.95 × 10^–12^	1.94 × 10^–09^	1.41 × 10^05^	2.14 × 10^05^	2.06 × 10^–07^	4.72 × 10^–04^
PentaF_HER2_	2.71 × 10^–12^	6.98 × 10^–09^	1.10 × 10^05^	1.28 × 10^05^	2.82 × 10^–07^	7.85 × 10^–04^

a Interactions were analyzed using biolayer interferometry (BLI) technique. Parameters of the interaction were determined by global fitting with the 2:1 “heterogeneous ligand” with ForteBio Data Analysis 11.0 software.

We used confocal microscopy to study whether BLI-assessed enhanced binding of multivalent variants to HER2 is also observed in the cellular context. Since monomeric Affibody_HER2:342_ displays no fluorescence, we chemically labeled Affibody_HER2:342_ with DyLight_550_ fluorescent dye and incubated Affibody_HER2:342_-DyLight_550_ with SKBR-3 cells on cold to prevent endocytosis. As shown in Figure 3E, Affibody_HER2:342_-DyLight_550_ stained the surface of SKBR-3, indicating efficient HER2 binding. Then, we incubated SKBR-3 cells with Affibody_HER2:342_-DyLight_550_ and with equimolar concentration of BiF_HER2_, TriF_HER2_, TetraF_HER2_, or PentaF_HER2_, and using confocal microscopy, we assessed the binding of particular HER2 ligands to the cell surface. We observed an almost complete loss of the red fluorescent signal of Affibody_HER2:342_-DyLight_550_ and concomitant appearance of strong green, fluorescent signal of BiF_HER2_, TriF_HER2_, TetraF_HER2_ or PentaF_HER2_ on the surface of SKBR-3 cells when monomeric HER2 ligand was mixed with multivalent variants (Figure 3E). These data confirm that the multivalency of developed HER2 ligands promotes their interaction with HER2 on the cell surface.

### 2.4 Efficient Clustering-Based Endocytosis of Multivalent HER2 Ligands

The effectiveness of anticancer therapy with cytotoxic conjugates relies on the highly efficient and selective delivery of cytotoxic drugs into cancer cells through receptor-mediated endocytosis. To investigate the internalization of multivalent HER2 ligands, we employed quantitative confocal microscopy using the high content Opera Phenix Plus platform and the inherent fluorescence of developed proteins. To this end, SKBR-3 cells were incubated with BiF_HER2_, TriF_HER2_, TetraF_HER2_, or PentaF_HER2_ for 30 min at 37 °C to allow for endocytosis, cytoplasm was then stained with CellMask reagent, and the intensity of the intracellular fluorescent punctate signal of particular multivalent HER2 ligand was measured in at least 200 individual cells. As demonstrated in Figure 4A, most of the fluorescent signal of BiF_HER2_ was detected on the surface of SKBR-3 cells, which agrees with the high resistance of HER2 to endocytosis. Interestingly, ligands with higher valency showed significantly enhanced cellular uptake in relation to BiF_HER2_, with the most efficient endocytosis observed for the tetravalent variant TetraF_HER2_ (Figure 4A). Due to superior binding and endocytosis, we decided to focus on TetraF_HER2_ in subsequent studies.

**Figure 4:**
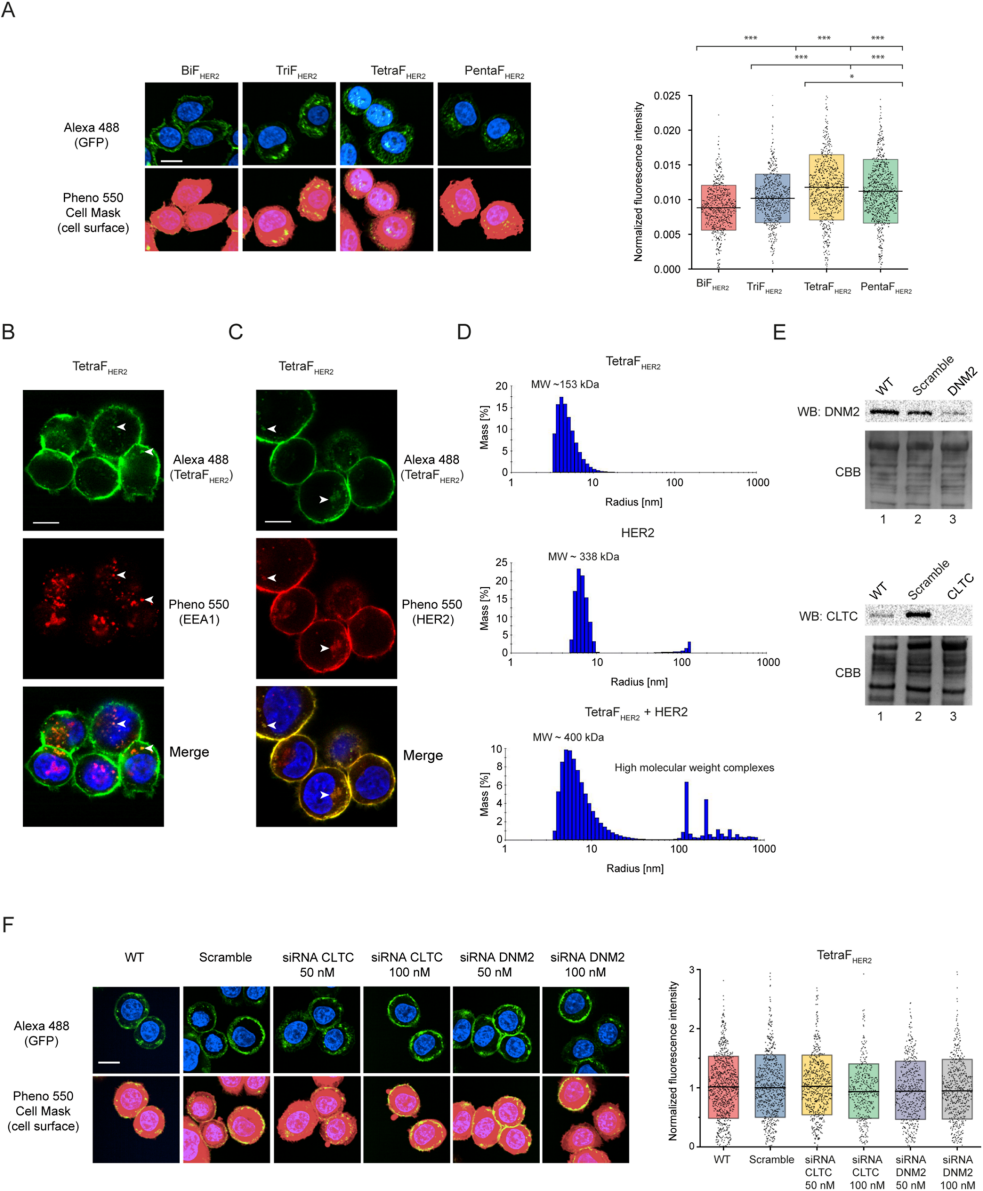
Efficient clustering-based endocytosis of multivalent HER2 ligands. (A) The internalization of BiF_HER2_, TriF_HER2_, TetraF_HER2_, and PentaF_HER2_ into SKBR-3 cells was analyzed by using quantitative confocal microscopy. Cells were treated with oligomers for 30 min at 37 °C. Nuclei were stained with NucBlue Live dye, and the cells were fixed, permeabilized with 0.1% Triton in PBS, and stained with HCS CellMask Deep Red Stain. Representative images from three independent experiments are shown. The scale bar represents 20 μm. Each gray spot in the graph represents the relative intracellular punctate signal intensity oligomers in the single cell. At least 200 cells for each condition from three independent experiments were measured. Horizontal lines in the graph represent the average intensity of the intracellular oligomer punctate signal, whereas boxes represent ± SD. Statistical analyses were performed using analysis of variance (ANOVA) with Tukey HSD for unequal N (Spjotvoll/Stoline) posthoc test (*p < 0.05; **p < 0.005 and ***p < 0.001). (B) Colocalization of TetraF_HER2_ with early endosome marker EEA1. SKBR-3 cells were incubated with TetraF_HER2_ for 30 min at 37 °C. Early endosomes were detected with rabbit polyclonal antibody specific for early endosome antigen 1 (EEA1) and antirabbit IgG secondary antibody conjugated to Alexa Fluor 594 (red). Scale bars are 20 μm. (C) Colocalization of TetraF_HER2_ with HER2. SKBR-3 cells were incubated with TetraF_HER2_ for 30 min at 37 °C. HER2 was detected with mouse monoclonal antibody specific for HER2 (ErbB2/HER2) and antimouse IgG secondary antibody conjugated to Alexa Fluor 594 (red). Scale bars represent 20 μm. (D) DLS signals of TetraF_HER2_, HER2, and mixtures of these proteins. DLS-estimated MW of the proteins are shown. High molecular weight complexes are seen upon incubation of TetraF_HER2_ and HER2. (E) Western blotting analysis of cell lysates of SKBR-3 cells treated with siRNA against clathrin heavy chain (CLTC), dynamin-2 (DNM2), and scramble siRNA as a control. CBB was used as a loading control. (F) Analysis of the effect of the depletion of CLTC and DNM2 on the endocytosis of TetraF_HER2_. SKBR-3 cells after CLTC and DNM2 knock-down were incubated with TetraF_HER2_ for 30 min at 37 °C, and internalization was analyzed using quantitative confocal microscopy. Representative images from three independent experiments are shown. The scale bar represents 20 μm. Each gray spot in the graph represents the relative intracellular punctate signal intensity of the TetraF_HER2_ in the single cell. At least 200 cells for each condition from three independent experiments were measured. Horizontal lines in the graph represent the average intensity of the intracellular TetraF_HER2_ punctate signal, whereas boxes represent ± SD. Statistical analyses were performed using analysis of variance (ANOVA) with Tukey HSD for unequal N (Spjotvoll/Stoline) posthoc test (*p < 0.05; **p < 0.005 and ***p < 0.001).

To confirm that the intracellular TetraF_HER2_ signal indeed represents an endocytosed protein, we studied the colocalization of TetraF_HER2_ with the marker of early endosomes, EEA1 and the marker of lysosomes, LAMP1 in SKBR-3 cells. The TetraF_HER2_ signal partially colocalized with both analyzed markers, indicating that after selective binding to HER2+ breast cancer cells, TetraF_HER2_ traffics *via* endosomes to lysosomes (Figures 4B and S2). To confirm that selective binding of TetraF_HER2_ to cell surface HER2 results in receptor-mediated endocytosis of TetraF_HER2_/HER2 complex, we also confirmed colocalization of TetraF_HER2_ with HER2 in intracellular puncta (Figure 4C). Additionally, we used cells without TetraF_HER2_ treatment and demonstrated that the signal of HER2 was detected mainly on the cell surface (Figure S3). These data indicate that HER2 internalization is forced by the presence of a multivalent ligand.

Recent reports indicated that cross-linking of cell surface receptors might promote their internalization, which occurs *via* aggregation-dependent endocytosis (ADE) or by simultaneous engagement of several endocytic pathways.^32,34^ To study whether TetraF_HER2_ triggers the clustering of HER2, we used dynamic light scattering (DLS). As shown in Figure 4D, the binding of TetraF_HER2_ to HER2 resulted in the formation of high molecular weight complexes, which confirms its capability to cluster HER2. Since the recently discovered ADE pathway is clathrin- and dynamin-independent, we employed siRNA technology to successfully knock-down clathrin heavy chain (CLHC) or dynamin-2 in SKBR-3 (Figure 4E). We then measured the effect of CLHC and dynamin-2 depletion on TetraF_HER2_ endocytosis using quantitative confocal microscopy. As shown in Figure 4F, the downregulation of both studied proteins did not affect the cellular uptake of TetraF_HER2_.

These data indicate that developed HER2 ligands, especially TetraF_HER2_, due to multivalency, trigger HER2 clustering, which in turn results in the promotion of highly efficient HER2 endocytosis *via* ADE.

2.5. Engineering of the high affinity, highly internalizing inherently fluorescent cytotoxic conjugate efficiently eliminates HER2+ breast cancer cells. We selected TetraF_HER2,_ characterized by its high stability, high affinity for HER2, and very effective and selective HER2-dependent endocytosis as a drug carrier for the engineering of a fluorescent cytotoxic conjugate targeting HER2+ breast cancer cells. To ensure site-specific attachment of the cytotoxic payload to TetraF_HER2_ and control over the number of drug molecules attached to the proteinaceous carrier (DAR, drug-to-antibody ratio), we decided to use sortase A-mediated ligation. In this strategy, the cytotoxic drug, a potent tubulin-destabilizing agent, monomethyl auristatin E bearing valine-citruline linker (vcMMAE), is coupled to the GGGSC peptide derivative, which is then ligated to the C-terminal LPTEGG sequence of TetraF_HER2_ by sortase A enzyme, resulting in the tetravalent, HER2-specific cytotoxic drug TetraF_HER2_-vcMMAE, containing exactly four MMAE molecules (Figure 5A). To this end, we synthesized the GGGS-(O2Oc)_2_-C-NH_2_ peptide (Figure 5B) and conjugated it with the vcMMAE, resulting in GGGS-(O2Oc)_2_-C­(vcMMAE)-NH_2_ (Figure 5C). Then, conditions were established for sortase mediated covalent coupling of drug-bearing peptide to the C-terminus of TetraF_HER2_. The successful attachment of the drug to TetraF_HER2_ was confirmed with SDS-PAGE, seen as a change in the migration of TetraF_HER2_-vcMMAE in relation to the unconjugated TetraF_HER2_ (Figure 5D), with Western blotting and anti-MMAE antibodies (Figure 5E), and with mass spectrometry (Figure 5F). These data indicate the successful engineering of the intrinsically fluorescent tetravalent TetraF_HER2_-vcMMAE conjugate capable of promoting HER2 endocytosis by inducing its cell surface clustering.

**Figure 5:**
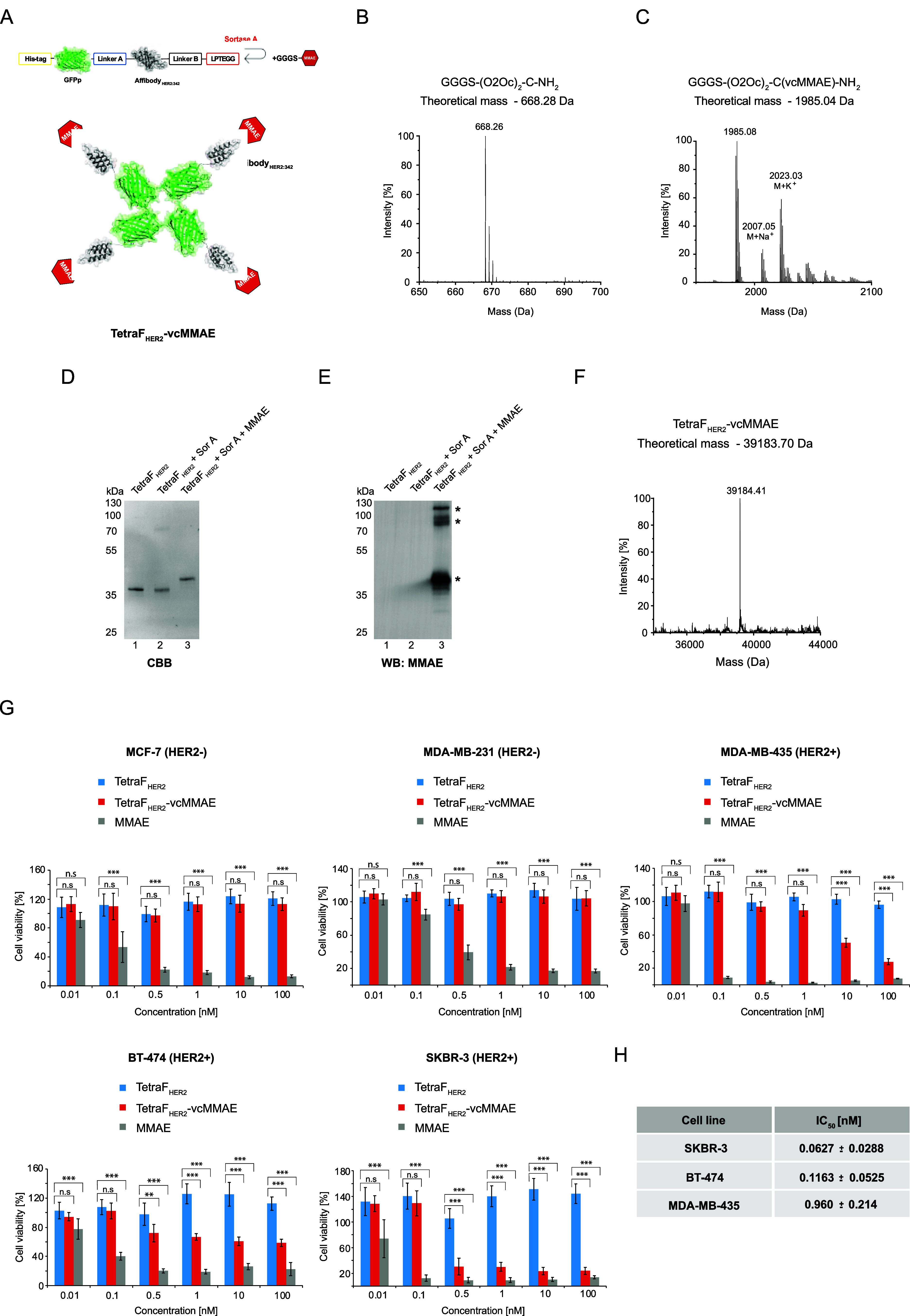
Development of an inherently fluorescent tetrameric cytotoxic conjugate against HER2-overexpressing breast cancer cells. (A) Schematic representation of the conjugation reaction and fluorescent tetrameric TetraF_HER2_-vcMMAE conjugate. Sortase A recognizes the LPETGG sequence within the tetrameric protein and mediates ligation of the peptide-linked MMAE, resulting in TetraF_HER2_-vcMMAE. The correctness of the synthesized GGGS-(O2Oc)_2_-C-NH_2_ peptide (B) and its conjugation with the vcMMAE (C) was confirmed with mass spectrometry. (D) The successful attachment of drug to TetraF_HER2_ was confirmed with SDS-PAGE, Western blotting, and anti-MMAE antibodies (E) and with mass spectrometry (F) asterisks in E mark nonfully denatured distinct oligomeric forms of the conjugate. (G) The cytotoxic effect of TetraF_HER2_-vcMMAE was tested on MCF-7, MDA-MB-231, MDA-MB-453, BT-474 and SKBR-3 cells. Cells were treated with increasing concentrations of TetraF_HER2_, TetraF_HER2_-vcMMAE, and free drug at 37 °C for 96 h. Cell viability was analyzed with the PrestoBlue Cell Viability Reagent. Data shown are the mean values of three independent experiments ± SD. Statistical analyses were performed with Kruskal–Wallis H test (*p < 0.05; **p < 0.005 and ***p < 0.001). H. The IC50 value of TetraF_HER2_-vcMMAE was calculated from Hill’s equation using Origin 7 software.

We assessed the toxicity of the TetraF_HER2_-vcMMAE for HER2+ and HER2- breast cancer cells using PrestoBlue Cell Viability Reagent. While free MMAE displayed high toxicity for HER2- MCF-7 and MDA-MB-231 cells, neither the TetraF_HER2_ scaffold alone nor the TetraF_HER2_-vcMMAE conjugate had any effects on the viability of these cells (Figure 5G). In contrast, TetraF_HER2_-vcMMAE was highly toxic for HER2+ SKBR-3, BT-474, and MDA-MB-453 cells, with an IC50 value of about 0.063 nM, 0.116 nM and 0.960 nM respectively (Figure 5H). We also studied the cytotoxicity and selectivity of TetraF_HER2_-vcMMAE with high-content confocal microscopy. To this end, SKBR-3 and MCF-7 cells were treated with free MMAE, TetraF_HER2_, or TetraF_HER2_-vcMMAE conjugate, NucBlue dye to stain all cells, and propidium iodide to label dead cells only, and cells were monitored with Opera Phenix Plus confocal microscope for up to 72 h with 6h intervals. Free MMAE caused the appearance of multiple propidium iodide-positive cells for both studied cell lines, as expected for nontargeted drugs (Figure 6). TetraF_HER2_ did not affect the viability of tested cells, whereas the TetraF_HER2_-vcMMAE conjugate induced the appearance of multiple propidium iodide signals only in HER2+ SKBR-3 breast cells and was fully neutral to MCF-7 breast cancer cells that are devoid of detectable HER2 expression (Figure 6). These data indicate that the intrinsically fluorescent tetravalent TetraF_HER2_-vcMMAE conjugate, by promoting aggregation-dependent endocytosis of HER2, serves as a highly selective and efficient drug carrier for targeted treatment of HER2+ breast cancer cells.

**Figure 6:**
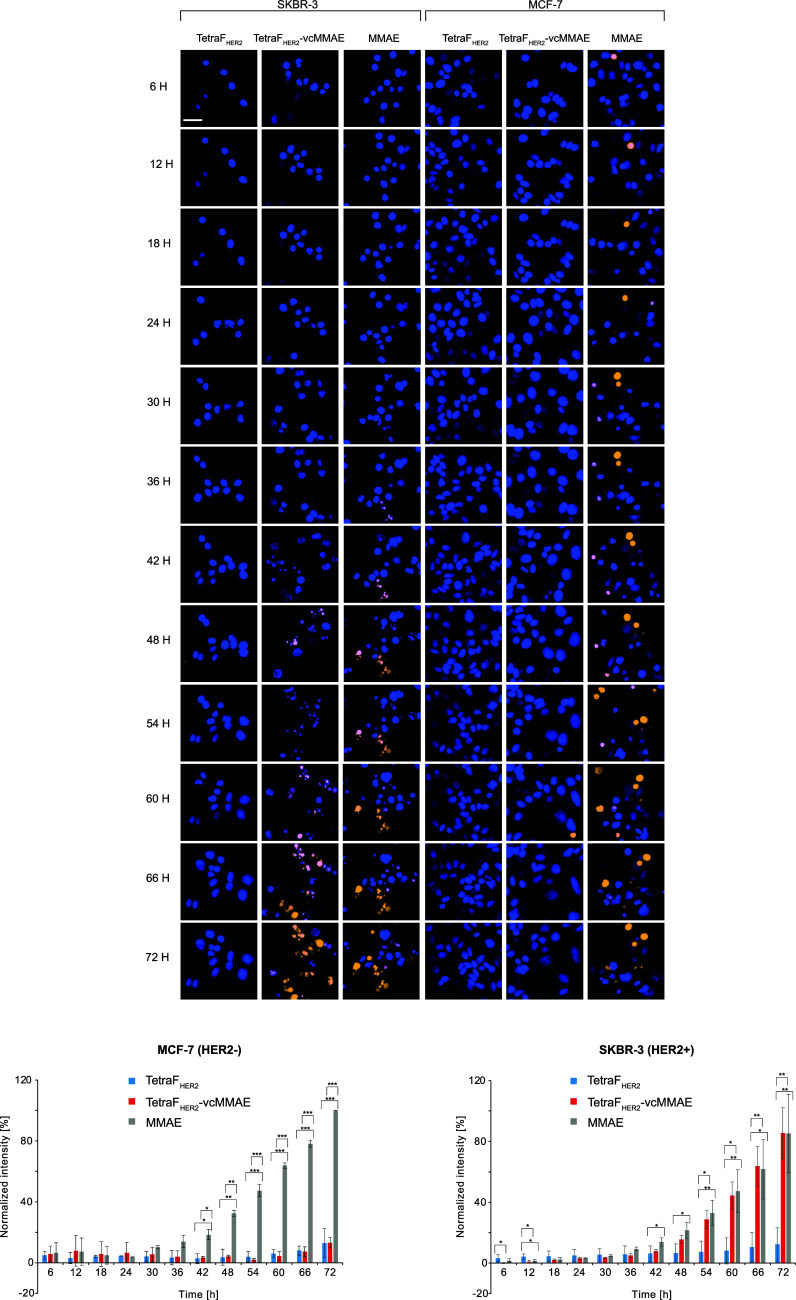
Microscopic analysis of the cytotoxic effect of TetraF_HER2_-vcMMAE. SKBR-3 and MCF-7 cells were treated with TetraF_HER2_, TetraF_HER2_-vcMMAE, and free drug at 37 °C for 72 h and imaged in real time using quantitative confocal microscopy. Dead cells were labeled with propidium iodide solution and nuclei of all cells were stained with NucBlue Live dye. Occasional pink color represents overlay of propidium iodide and NucBlue Live staining. Representative images from three independent experiments are shown. The scale bar represents 20 μm. The graph shows the normalized percentages of the intensities of the propidium iodide derived signals. Average values from at three independent experiments ± SD are shown. Statistical analyses were performed using analysis of variance (ANOVA) with the Fishers LSD posthoc test (*p < 0.05; **p < 0.005, and ***p < 0.001).

## 3 Discussion and Conclusions

Currently, one of the most promising strategies in breast cancer treatment is targeted therapy with cytotoxic conjugates. In this approach, the selective and highly efficient internalization of cytotoxic conjugates into cancer cells *via* receptor-mediated endocytosis is crucial for the efficacy of this therapy. The discovery of HER2, which is closely associated with aggressive breast cancer, and the demonstration that HER2 is a druggable therapeutic target led to the development and approval of several HER2-targeted drugs. So far, two HER2-specific ADCs, trastuzumab emtansine (T-DM1) and trastuzumab deruxtecan (T-DXd), have been approved by the FDA, but their efficacy is limited at least partially by their inefficient HER2-dependent internalization.^15,21,22^ Cancer cells can alter endocytic pathways as a defense mechanism, limiting the efficacy of the therapy.^37^ T-DM1 and T-DXd are composed of the bivalent antibody trastuzumab and are mostly internalized *via* clathrin-mediated endocytosis (CME). Therefore, they are susceptible to alterations in CME, which can be downregulated in cancer cells.^37^ Li et al. recently reported HER2-specific, biparatopic ADC, MEDI4276, which was characterized by enhanced internalization and potency in relation to T-DM1, implicating that the modular design of the drug carrier may elevate ADC action.^38^ Therefore, targeting molecules that will simultaneously engage multiple endocytic pathways is desirable for targeted therapies with cytotoxic conjugates. As HER2 is a poor internalizing receptor, novel strategies are needed to improve the endocytosis and lysosomal trafficking of HER2.

Several examples show that the clustering of the receptor on the cell surface by multivalent ligands increases the efficiency of receptor endocytosis.^33,34,39,40^ Multivalent protein drug conjugates (PDCs) are an attractive alternative to monovalent or bivalent cytotoxic conjugates. Multivalent ligands display an increased affinity for the cancer-relevant receptor.^41^ Due to the higher number of receptor binding sites, multivalent PDC more effectively recognizes the receptor on the cancer cell surface and forms a stable endocytic complex with it, which is required for efficient PDC internalization *via* receptor-dependent endocytosis.^41^ Our recent findings demonstrate that the clustering of fibroblast growth factor receptor 1 (FGFR1) with multivalent ligands essentially enhances receptor endocytosis efficiency by engaging multiple endocytic pathways at the same time.^34^ In addition, Paul et al. recently demonstrated that enhanced internalization of receptors, including HER2, triggered by multivalent ligands, occurs *via* aggregation-dependent endocytosis (ADE).^32^ Therefore, multivalent PDCs may represent a tool to increase receptor-dependent endocytosis and through selective drug delivery.

Here, using a very attractive, low molecular weight HER2 ligand, Affibody_HER2:342_, and a GFPp oligomerization scaffold, we developed novel, multivalent, inherently fluorescent HER2-specific ligands characterized by high stability, achieved likely by firmness of the fold of both GFPp and Affibody_HER2:342_. We confirmed that multivalent ligands are highly specific for HER2 and can recognize HER2 exposed on the cell surface. Our strategy for generating oligomeric ligands is simple regarding size and valence optimization. It is possible to obtain targeting molecules with more receptor binding sites. However, our previous studies show that too high valence can have an inhibitory effect on receptor endocytosis.

We selected TetraF_HER2,_ characterized by its high stability, high affinity for HER2 and the ability to cross-link HER2 to engineer a fluorescent cytotoxic conjugate. We confirmed that the fluorescent tetravalent TetraF_HER2_-vcMMAE conjugate, due to enhanced clustering-dependent endocytosis of HER2, efficiently and selectively killed HER2-overproducing breast cancer cells with IC50 values in the low picomolar range, which is comparable or even more potent than HER2-specific bivalent ADCs used in clinics: T-DM1 and T-DXd.^42,43^ At the same time TetraF_HER2_-vcMMAE displays minimal toxicity for HER2- cells, confirming highly selective HER2-dependent drug delivery into cancer cells The only one HER2-targeting multivalent ADC reported so far, the biparatopic MEDI4276, is a large and complex molecule based on a modified mAb scaffold that combines two distinct HER2 antigen binding sites.^38^ Despite its biparatopic architecture, MEDI4276 is capable of binding more than two HER2 molecules, resulting in HER2 clustering.^44^ In comparison to the biparatopic MEDI4276, the tetravalent conjugate TetraF_HER2_-vcMMAE developed by us contains four identical HER2 binding sites, ensuring highly efficient receptor clustering, is inexpensive to produce and further modify, and its stable, inherent fluorescence allows for selectivity assessment and precise tracking of the conjugate inside the cancer cells. This feature can be used in future studies to monitor drug biodistribution and therapeutic response *in vivo* or possibly can be used in imaging-guided cancer therapy of HER2+ breast cancer.

In summary, we have developed a novel tetravalent fluorescent cytotoxic conjugate TetraF_HER2_-vcMMAE, which can cluster HER2 and increase the efficiency of HER2 endocytosis *via* ADE. This approach may overcome HER2 immobility and improve the efficacy of cytotoxic conjugates targeting HER2 in breast cancer. The future studies should focus on evaluating the *in vivo* potential of TetraF_HER2_-vcMMAE for selective targeting of HER2+ breast cancer.

## 4 Experimental Section

### 4.1 Antibodies and Reagents

The primary antibodies directed against HER2 (ErbB2/HER2, #sc-33684), His-Tag (His-Probe, #sc-8036) and dynamin-2 (#sc-17807) were from Santa Cruz Biotechnology (Dallas, TX). Antitubulin primary antibody (#T6557) was from Sigma-Aldrich (St Louis, MO). Alexa fluor 594 goat antirabbit secondary antibody (#A11037) was from Thermo Fisher Scientific (Waltham, MA). The primary antibodies directed against LAMP1 (no. ab24170) and goat antimouse AF-594 secondary antibody (no. ab150120) were from Abcam (Cambridge, U.K.). The primary antibodies directed against clathrin heavy chain (no. 610499) were from BD Transduction Laboratories (Bergen, NJ). Anti-EEA1 primary antibody (no. 2411S) was from Cell Signaling (Danvers, MA). HRP-conjugated secondary antibodies were obtained from Jackson Immuno-Research Laboratories (Cambridge).

Reagents used for the solid-phase peptide synthesis were as follows: Amino Fmoc-Gly-OH, Fmoc-l-Ser­(*t*Bu)–OH, and Fmoc-O2Oc-O2Oc–OH; DIC/Oxyma Pure, EDT (ethane-1,2-dithiol), piperidine, TIS (triisopropylsilane), DIPEA (*N,N*-diisopropylethylamine), DMF (*N,N*-dimethylformamide), DCM (dichloromethane), and TFA (trifluoroacetic acid) were purchased from Iris Biotech GmbH (Marktredwitz, Germany). HPLC-grade ACN (acetonitrile) and Et_2_O (diethyl ether) were obtained from Avantor (Gliwice, Poland). Fmoc-Cys-RAM Tenta Gel was from Rapp Polymere GmbH (Tübingen, Germany). The cytotoxic agents, MMAE (monomethyl auristatin E) and mc-vc-PAB–MMAE (#HY-15575) were from MedChemExpress (Monmouth Junction, NJ).

The NucBlue Reagent (Hoechst 33342) (#R37605), DyLight 550 NHS Ester (#62263) and HCS CellMask Stain Deep Red (#32721) were from Thermo Fisher Scientific. Propidium iodide (#25535–16–4) was from Sigma-Aldrich (St Louis, MO).

### 4.2 Cells

The cell lines SKBR-3, MCF-7, BT-474, MDA-MB-453, and MDA-MB-231 were obtained from the American Type Culture Collection (ATCC) (Manassas, VA). SKBR-3, MCF-7, MDA-MB-453 and MDA-MB-231 cell lines were cultured in 5% CO_2_ atm at 37 °C in Dulbecco’s modified Eagle’s medium (Biowest, Nuaille, France) supplemented with 10% fetal bovine serum (FBS) (Thermo Fisher Scientific, Waltham, MA) and antibiotics mix (100 U/mL penicillin and 100 μg/mL streptomycin) (Thermo Fisher Scientific, Waltham, MA). BT-474 cell line was cultured in the Hybri-Care Medium (ATCC, Manassas, VA) supplemented with 1.5 g/L sodium bicarbonate, 10% FBS and antibiotics mix. All cell lines were seeded onto tissue culture plates 1 day prior to the start of the experiments.

### 4.3 siRNA Transfection

According to the manufacturer’s instructions, cells were transfected with siRNA against endocytic proteins with DharmaFECT (Horizon, Cambridge, U.K.). SKBR-3 cells were treated with siRNA against clathrin heavy chain CLTC (no. 4390824, Thermo Fisher Scientific, Waltham, MA) and dynamin-2 DNM2 (no. 4390824, Thermo Fisher Scientific, Waltham, MA) at a concentration of 50 or 100 nM for 24 h. Control cells were transfected with 100 nM nontargeting siRNA (#D-001810–01–50, Horizon, Cambridge, U.K.). After 24 h, the transfection medium was replaced with a complete medium and the incubation was continued for another 24 h. Then, cells were seeded on microscope plates (7000 cells per well) in a complete medium and left to attach overnight. The next day, the cells were analyzed using confocal microscopy. The effectiveness of CLTC and DNM2 downregulation was confirmed by Western blotting.

### 4.4 Recombinant Proteins

The coding sequence of GFPp_Affibody_HER2:342_ with an N-terminal His-Tag and a C-terminal SGGSGGSGGSGGLPETGG motif in pET3d and monomeric Affibody_HER2:342_ in pET3d were obtained as a custom gene synthesis from Gene Universal (Newark, DE). The proteins were expressed in the BL21 CodonPlus (DE3)-RIL strain (Agilent Technologies, Santa Clara, CA). The bacterial cultures were grown at 37 °C until OD_600_ = 0.4 and then at 16 °C to OD_600_ = 0.8. Protein expression was induced by the addition of 1 mM IPTG, followed by incubation at 16 °C for 16 h. Proteins were purified by affinity chromatography using Ni–NTA resin. The purity and identity of obtained proteins were confirmed by SDS-PAGE and Western blotting.

Human HER2 receptor with the Fc Tag (#HE2-H5253) was obtained from AcroBiosystems (Newark, DE). Evolved sortase A (eSortA) pentamutant with improved kinetics and activity was produced in an as described earlier.^45,46^

### 4.5 Isolation of Various Oligomeric Forms

GFPp_Affibody_HER2:342_ oligomers were separated under nondenaturing conditions by Native PAGE. The mixture of proteins was separated on 10% native gels using a Tris-Glycine Running Buffer (25 mM Tris·HCl, 192 mM glycine, pH 8.3). Native gels were run on ice, and after the electrophoretic separation, the bands representing the individual oligomers were cut out under UV light and transferred to a buffer containing 20 mM Tris·HCl, 150 mM glycine, 0.02% SDS, pH 8.3 with shaking for 48 h at 4 °C. Next, oligomers were transferred to the 25 mM Na^+^-HEPES pH 8.0, 300 mM NaCl, 1 mM EDTA, 1 mM DTT, and 5% glycerol using HiPrep 26/10 Desalting Column (Thermo Fisher Scientific, Waltham, MA).

### 4.6 Size Exclusion Chromatography

The oligomeric state of the purified proteins was assessed by size exclusion chromatography (SEC) using a KTA explorer FPLC system with HiLoad Superdex 75 HR 10/300 GL or Superdex 200 10/300 GL columns. The oligomers at a concentration of 1 mg/mL were injected into a column and run at a flow rate of 1 mL/min in PBS buffer. The absorbance spectra were monitored at 280 nm. Molecular weight standards containing BPTI, cytochrome C, carbonic anhydrases, human serum albumin, α-lactoglobulin, chymotrypsinogen A, and ovalbumin (Sigma-Aldrich, St. Louis, MO) were used to generate a standard curve from which the average molecular weights of individual oligomers were calculated.

### 4.7 Analysis of Protein Stability

To analyze the stability of the proteins, the oligomers (20 μg) were incubated in a serum-free medium at 37 °C for 96 h. At distinct time points (0, 24, 48, 72, and 96 h), samples were taken and the proteins were analyzed with Native PAGE, SDS-PAGE, and Western blotting. The stability of the proteins was also analyzed by measuring their fluorescence. Proteins at a concentration of 1 μM were incubated in 10-fold diluted human serum at 37 °C for 96 h. Fluorescence spectra were acquired using an FP-8500 spectrofluorometer (Jasco, Japan) with excitation at 488 nm and emission in the 500–650 nm range.

### 4.8 BLI Measurements

Binding analysis of monomeric Affibody_HER2:342_ and oligomeric variants of GFPp_Affibody_HER2:342_ to HER2-Fc was performed using biolayer interferometry (BLI) with ForteBio Octet K2 (Pall ForteBio, San Jose, CA). HER2-Fc (10 μg/mL) was immobilized on Protein A sensors, and the association and dissociation phases were monitored at various protein concentrations (25, 50, and 100 nM) in a PBS buffer. A reference sensor without HER2-Fc was used as a control. Kinetic parameters of the interaction were determined based on a global 2:1 “heterogeneous ligand” fitting using ForteBio Data Analysis 11.0 software (Pall ForteBio, San Jose, CA).

### 4.9 Fluorescence Microscopy

Binding analysis of oligomeric variants of GFPp_Affibody_HER2:342_ to HER2 was performed by using SKBR-3 (HER2-positive) and MCF-7 (HER2-negative) cell lines. Cells (7 000 cells per well) were incubated with the oligomers at a concentration of 300 nM for 30 min on ice. Next, cells were washed with PBS, the nuclei were stained with NucBlue Live dye, and the cells were fixed in a 4% paraformaldehyde solution.

To test whether oligomeric proteins bind more tightly to the HER2 receptor on the cell surface than the monomeric protein, SKBR-3 cells were preincubated with 300 nM DyLight 550-labeled monomeric Affibody_HER2:342_ for 10 min on ice. Next, GFPp_Affibody_HER2:342_ oligomers (300 nM) were added, and incubation was continued for 30 min. Cells incubated with monomeric protein only were used as a control. Cells were washed with PBS, and the nuclei were stained with the NucBlue Live dye. Cells were fixed in 4% paraformaldehyde solution, permeabilized with 0.1% Triton in PBS, and stained with HCS CellMask Deep Red Stain.

To analyze the internalization of GFPp_Affibody_HER2:342_ oligomers by cells expressing HER2, SKBR-3 cells were incubated with 300 nM oligomers in a serum-free medium at 37 °C for 30 min. After this time, the internalization was stopped by cooling down cells on ice. Cells were subsequently washed with PBS and nuclei were stained with NucBlue Live dye. Cells were fixed in 4% paraformaldehyde solution, permeabilized with 0.1% Triton X-100 in PBS, and stained with HCS CellMask Deep Red Stain.

Fixed and labeled cells were analyzed with quantitative confocal microscopy using the Opera Phenix Plus High-Content Screening System (PerkinElmer, Waltham, MA). Measurements were carried out using confocal mode with 63× Water, NA 1.15 objective with binning 2 using two peaks autofocus. 37 fields per well were imaged, with 6–8 Z-stack per field at 0.5 μm intervals to ensure comprehensive imaging of the cell. 2160 × 2160 px Camera ROI was used to capture the images. The Harmony High-Content Imaging and Analysis Software (version 5.1; PerkinElmer, Waltham, MA) was used for image acquisition and analysis. The number of cells and the cell boundaries were determined using DAPI and CellMask Deep Red, respectively. The intensity in fluorescent signal in Alexa488 channel was measured and calculated between cells. Images were assembled in Illustrator (Adobe) with only linear contrast and brightness adjustments.

To analyze the colocalization of TetraF_HER2_ with an early endosome marker protein (EEA1) and HER2 receptor, SKBR-3 cells were incubated with 300 nM TetraF_HER2_ for 30 min at 37 °C. To confirm the colocalization of TetraF_HER2_ with lysosome marker (LAMP1), cells were incubated with tetrameric protein for 8 h. Cells were subsequently washed with PBS and nuclei were stained with NucBlue Live dye. Cells were fixed in 4% paraformaldehyde, permeabilized with 0.1% Triton X-100 and blocked with 2% BSA for 30 min. Next, cells were incubated with primary antibodies against EEA1, HER2 or LAMP1 respectively at 4 °C overnight. Then, cells were washed with PBS and incubated with secondary antibody (goat antirabbit IgG secondary antibody conjugated to Alexa Fluor 594 or with goat antimouse AF-594 secondary antibody) for 1 h at RT. Fixed and labeled cells were analyzed with confocal microscopy using the STELLARIS Confocal Microscope Platform (Leica, Wetzlar, DE). Measurements were carried out using confocal mode with 86× Water objective. The Leica LasX Software was used for image acquisition and analysis. Images were assembled in Fiji and Illustrator (Adobe) with only linear contrast and brightness adjustments.

### 4.10 Synthesis of GGGS-(O2Oc)_2_-C­(vcMMAE)-NH_2_

The GGGS-(O2Oc)_2_-C-NH_2_ peptide was synthesized utilizing the Fmoc SPPS strategy on the Fmoc-Cys-RAM Tenta Gel resin (Rapp Polymere, Germany). Amino acid coupling using DIC with Oxyma Pure was performed on a microwave peptide synthesizer (CEM Liberty Blue 2.0) using standard microwave power and coupling times. The finished peptide was cleaved from the resin using a mixture of TFA/TIPS/1,2-EDT/thioanisole/thiophenol/H_2_O (90:2:2:2:2:2). Next, the majority of TFA was evaporated by using a stream of nitrogen. Finally, the peptide was precipitated with cold Et_2_O and centrifuged. Several Et_2_O washing and centrifugation cycles were performed to ensure that the pellets had a minimal amount of scavenging reagents. The peptide purification was performed on Waters 1525 HPLC system using Phenomenex Gemini-NX C18 column utilizing a gradient of MeCN in 0.1% TFA. The identity of the peptide was confirmed by mass spectrometry (Bruker ESI-Q-ToF Compact). The calculated mass was 668.28, found 668.26. The pooled peptide fractions were frozen and lyophilized. GGGS-(O2Oc)_2_-C-NH_2_ (36.9 mg, 55.2 μmol) and maleimide-vcMMAE (MC-vc-PAB–MMAE, 21.8 mg, 16.6 μmol, 0.3 equiv), both dissolved in DMAc, were mixed (418 uL), followed by the addition of DIPEA (28.8 μL, 165.6 μmol, 3 equiv). The reaction was conducted at RT for 1 h. The solvent was then removed under a vacuum, and the GGGS-(O2Oc)_2_-C­(vcMMAE)-NH_2_ was purified by RP-HPLC and lyophilized. The identity of the product was confirmed by mass spectrometry.

### 4.11 Conjugation of the 4 × GFPp_ Affibody_HER2:342_ with MMAE *via* Sortase A-Mediated Ligation

Purified engineered tetrameric GFPp_Affibody_HER2:342_ containing the C-terminal LPETGG sequence was transferred to the sortase A reaction buffer (25 mM Na^+^-HEPES pH 7.6, 154 mM NaCl, 5 mM CaCl_2_, 2 mM TCEP) using Zeba Spin Desalting Columns (Thermo Fisher Scientific, Waltham, MA). The final concentration of protein used in the conjugation reaction was 250 μg/mL. The GGGS-(O2Oc)_2_-C­(vcMMAE)-NH_2_ peptide was added to the protein solution to a final concentration of 891 μM. Then, sortase A was added to a final concentration of 5 μM, and the mixture was incubated overnight at 16 °C with 550 rpm end-overend rotation. After incubation, the reaction mixture was subjected to size exclusion chromatography in PBS using a Superdex 75 10/300 GL column (GE Healthcare, Piscataway, NJ) to remove unconjugated peptide and sortase A. The identity of the conjugate was confirmed by mass spectrometry.

### 4.12 Cytotoxicity Assay

The cytotoxicity of the 4 × GFPp_Affibody_HER2:342_MMAE was tested on the HER2-negative cell line (MCF, MDA-MB-231) and HER2-positive cell line (SKBR-3, BT-474, and MDA-MB-453). Cells in the appropriate complete medium were plated at 5000 cells per well in 96-well plates and incubated overnight at 37 °C in the presence of 5% CO_2_. Cells were treated with increasing concentrations (from 0.01 to 100 nM) of 4 × GFPp_Affibody_HER2:342_ (negative control), 4 × GFPp_Affibody_HER2:342_MMAE or free drug – MMAE (positive control) for 96 h at 37 °C. Next, according to the manufacturer’s protocol, cell viability was measured using PrestoBlue Cell Viability Reagent (#A13262, Thermo Fisher Scientific, Waltham, MA). Fluorescence emission at 590 nm (excitation at 560 nm), reflecting the viability of the cells, was measured using an Infinite M1000 PRO plate reader (Tecan, Männedorf, Switzerland). Statistical analyses were performed for three independent experiments, and IC50 values were calculated based on the Hill equation using Origin 7 software (Northampton, MA).

The cytotoxic effect of 4 × GFPp_Affibody_HER2:342_MMAE was also analyzed by using confocal microscopy. SKBR-3 and MCF-7 cells (7000 cells per well) were treated with tetrameric conjugate, unconjugated protein, and free drug MMAE (concentration 0.5 nM) at 37 °C for 72 h and imaged in real time using confocal microscopy. Propidium iodide solution (1 ul of 1 mg/mL stock solution) as added to visualize dead cells and NucBlue Live (1 μL) was added to live cells. Cells were analyzed with the quantitative confocal microscopy using the Opera Phenix Plus High-Content Screening System. Measurements were carried out using confocal mode with 20× Water, NA 1.15 objective with binning 2 using two peaks autofocus. The 2160 × 2160 px Camera ROI was used to capture the images. The Harmony High-Content Imaging and Analysis Software (version 5.1; PerkinElmer, Waltham, MA) was used for image acquisition and analysis. Number of cells and the cells undergoing apoptosis were determined using the DAPI and propidium iodide respectfully.

### 4.13 Mass Spectrometry

Intact protein LC-MS was carried out on an M-Class Acquity UPLC system coupled to a Synapt XS HRMS equipped with an ESI ion source interface. Mobile phase A consisted of H_2_O + 0.1% formic acid (FA), 0.05% TFA, while mobile phase B consisted of ACN + 0.1% FA, 0.05% TFA. Approximately 2–5 pmoles of protein were injected, desalted on-system, and a 5 min 20–80% B linear gradient was applied for sample separation on a nanoEase M/Z BEH C4 300 Å, 5 μm, 300 μm × 50 mm column, which was kept at 80 °C. MS data was collected at 1 scan/s through a 300–3000 *m*/*z* range in positive polarity and TOF resolution mode. Glufibrinopeptide B solution was acquired in the reference function, and the correction was applied in acquisition. Raw data was processed using the MassLynx V4.2 software. The protein peak from each run was integrated, and the combined spectra were subtracted from the background and then deconvoluted using the MaxEnt1 algorithm.

## Supplementary Material



## Data Availability

Original data used for preparation of this manuscript have been deposited in the Zenodo repository and is available under following doi:10.5281/zenodo.15050603.

## Notes

Purification of GFPp_Affibody_HER2:342_ and monomeric Affibody_HER2:342;_ colocalization of TetraF_HER2_ with lysosomes and colocalization of TetraF_HER2_ with HER2 (PDF)

## Abbreviations Used

ACN: acetonitrile
ADE: aggregation-dependent endocytosis
ADC: antibody-drug conjugate
AKT: protein kinase B
BLI: biolayer interferometry
CLHC: clathrin heavy chain
CME: clathrin-mediated endocytosis
DCM: dichloromethane
DAR: drug-to-antibody ratio
DIPEA: *N*,*N*-diisopropylethylamine
DLS: dynamic light scattering
DMF: *N*,*N*-dimethylformamide
EEA1: early endosome antigen 1
EDT: ethane-1,2-dithiol
EGFR: epidermal growth factor receptor family
FGFR1: fibroblast growth factor receptor 1
GFPp: green fluorescent protein polygons
HER2: human epidermal growth factor receptor 2
Hsp90: heat shock protein 90
MAPK: mitogen-activated protein kinase
MMAE: monomethyl auristatin E
mTOR: mammalian target of rapamycin
PI3K: phosphatidylinositol 3-kinase
PDC: protein drug conjugate
RTK: receptor tyrosine kinase
SEC: size exclusion chromatography
T-DM1: trastuzumab emtansine
T-DXd: trastuzumab deruxtecan
TFA: trifluoroacetic acid
TIS: triisopropylsilane
TNBC: triple-negative breast cancer
