# Supplementary material for: Innately Fluorescent Tetravalent Cytotoxic Conjugate TetraFHER2-vcMMAE Engages Aggregation-Dependent Endocytosis of HER2 for Enhanced Intracellular Drug Delivery
Source: J Med Chem. 2025 Jun 29;68(13):13872–86. doi: 10.1021/acs.jmedchem.5c00782 (PMC12257533; doi:10.1021/acs.jmedchem.5c00782)

Supporting Information

The innately fluorescent tetravalent cytotoxic conjugate TetraF$_{HER2}$-vcMMAE engages aggregation-dependent endocytosis of HER2 for enhanced intracellular drug delivery

Natalia Porębska[1]*, Aleksandra Chorążewska[1], Krzysztof Ciura[1], Adam Pomorski[2], Artur Krężel[2] and Łukasz Opaliński[1]*

[1]Department of Medical Biotechnology, Faculty of Biotechnology, University of Wroclaw, F. Joliot-Curie 14a, 50-383 Wroclaw, Poland

[2]Department of Chemical Biology, Faculty of Biotechnology, University of Wroclaw, F. Joliot-Curie 14a, 50-383 Wroclaw, Poland

*Correspondence should be addressed to N.P (natalia.porebska@uwr.edu.pl) and Ł.O (lukasz.opalinski@uwr.edu.pl)

Table of Contents:

1. Figure S1. Purification of GFPp_Affibody$_{HER2:342}$ and monomeric Affibody$_{HER2:342}$ (Page 2)
2. Figure S2. Co-localization of TetraF$_{HER2}$ with the lysosomes (Page 2)
3. Figure S3. Co-localization of TetraF$_{HER2}$ with HER2 (Page 3)
4. Original data for Figure 1 (Page 4)
5. Original data for Figure 2 (Page 5)
6. Original data for Figure 3 (Page 6)
7. Original data for Figure 4 (Page 7)
8. Original data for Figure 5 (Page 8)

Fig. S1

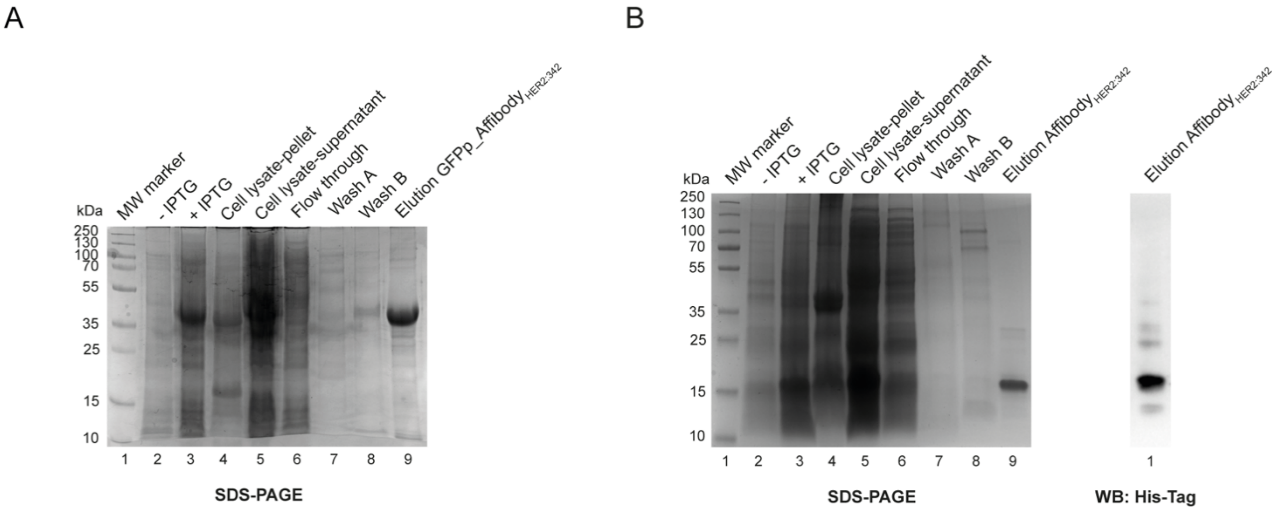

Fig. S1. Purification of GFPp_Affibody$_{HER2:342}$ and monomeric Affibody$_{HER2:342}$. A. The mixture of GFPp_Affibody$_{HER2:342}$ oligomers was purified by affinity chromatography and analyzed using SDS-PAGE. **B**. The monomeric ligand Affibody$_{HER2:342}$ was purified by affinity chromatography and analyzed using SDS-PAGE and western blotting with anti-His-Tag antibodies.

Fig. S2

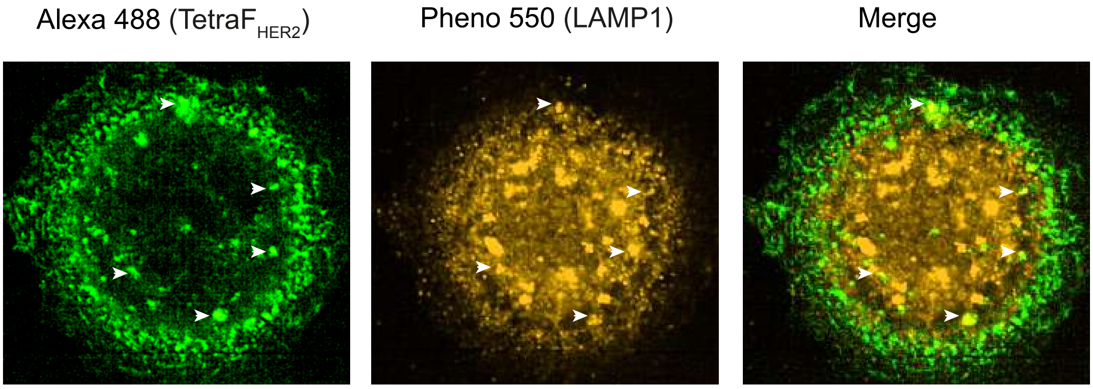

Fig. S2. Co-localization of TetraF$_{HER2}$ with the lysosomes. SKBR-3 cells were incubated with TetraF$_{HER2}$ for 8 h at 37°C. Lysosomes were detected with rabbit antibody Lamp1 and anti-rabbit IgG secondary antibody conjugated to Alexa Fluor 594 (red). Fixed and labeled cells were analyzed with quantitative confocal microscopy using the Opera Phenix Plus High-Content Screening System (Perkin Elmer, Waltham, MA, USA). Measurements were

carried out using confocal mode with 63 × Water, NA 1.15 objective with binning 2 using two peaks autofocus. 2160 × 2160 px Camera ROI was used to capture the images. The Harmony High-Content Imaging and Analysis Software (version 5.1; Perkin Elmer, Waltham, MA, USA) was used for image acquisition and analysis.

Fig. S3

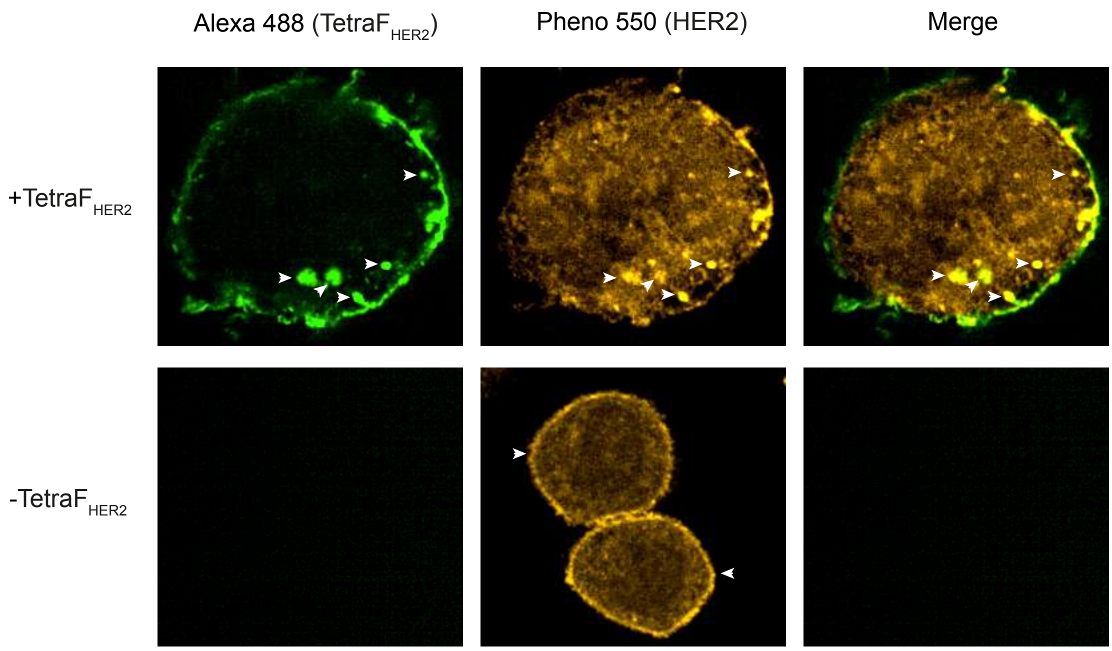

Fig. S3. Co-localization of TetraF$_{HER2}$ with HER2. SKBR-3 cells were incubated with TetraF$_{HER2}$ for 30 min at 37°C. Cells non treated with TetraF$_{HER2}$ were used as a control. HER2 receptor was detected with mouse monoclonal antibody specific for HER2 (ErbB2/HER2) and anti-mouse IgG secondary antibody conjugated to Alexa Fluor 594 (red). Fixed and labeled cells were analyzed with quantitative confocal microscopy using the Opera Phenix Plus High-Content Screening System (Perkin Elmer, Waltham, MA, USA). Measurements were carried out using confocal mode with 63 × Water, NA 1.15 objective with binning 2 using two peaks autofocus. 2160 × 2160 px Camera ROI was used to capture the images. The Harmony High-Content Imaging and Analysis Software (version 5.1; Perkin Elmer, Waltham, MA, USA) was used for image acquisition and analysis.

Original data for Figure 1

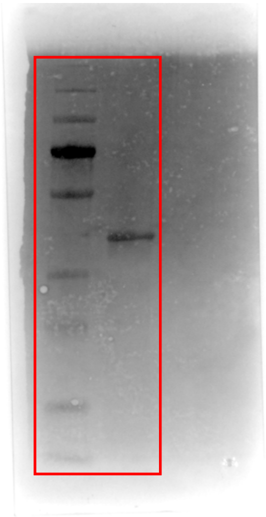

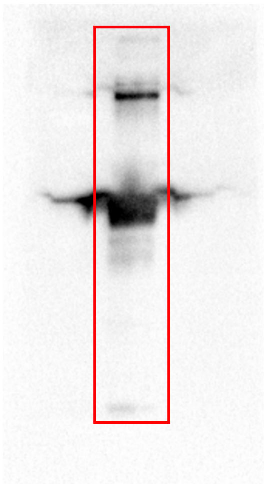

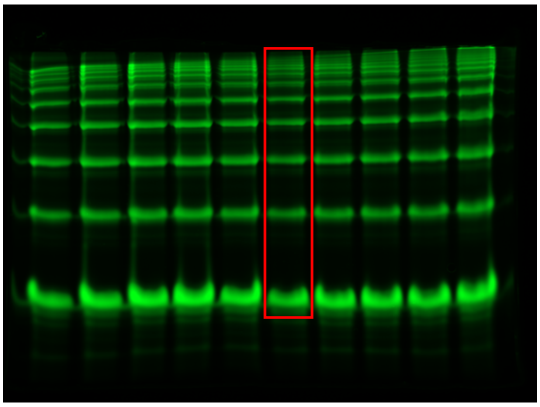

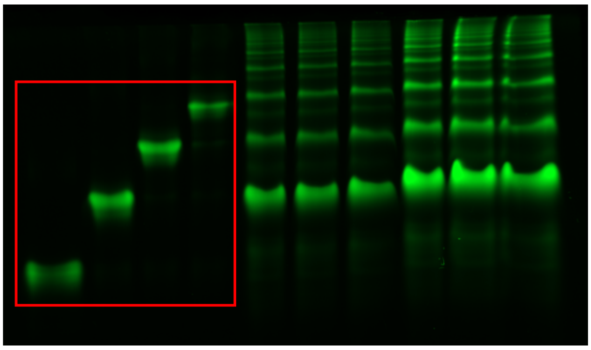

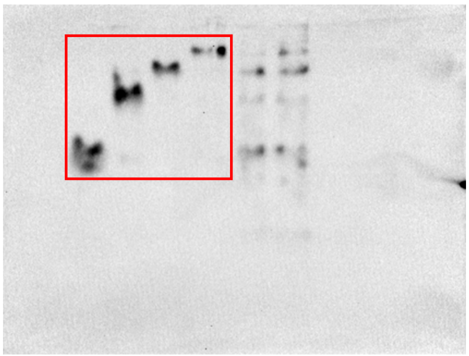

Original data for Figure 2

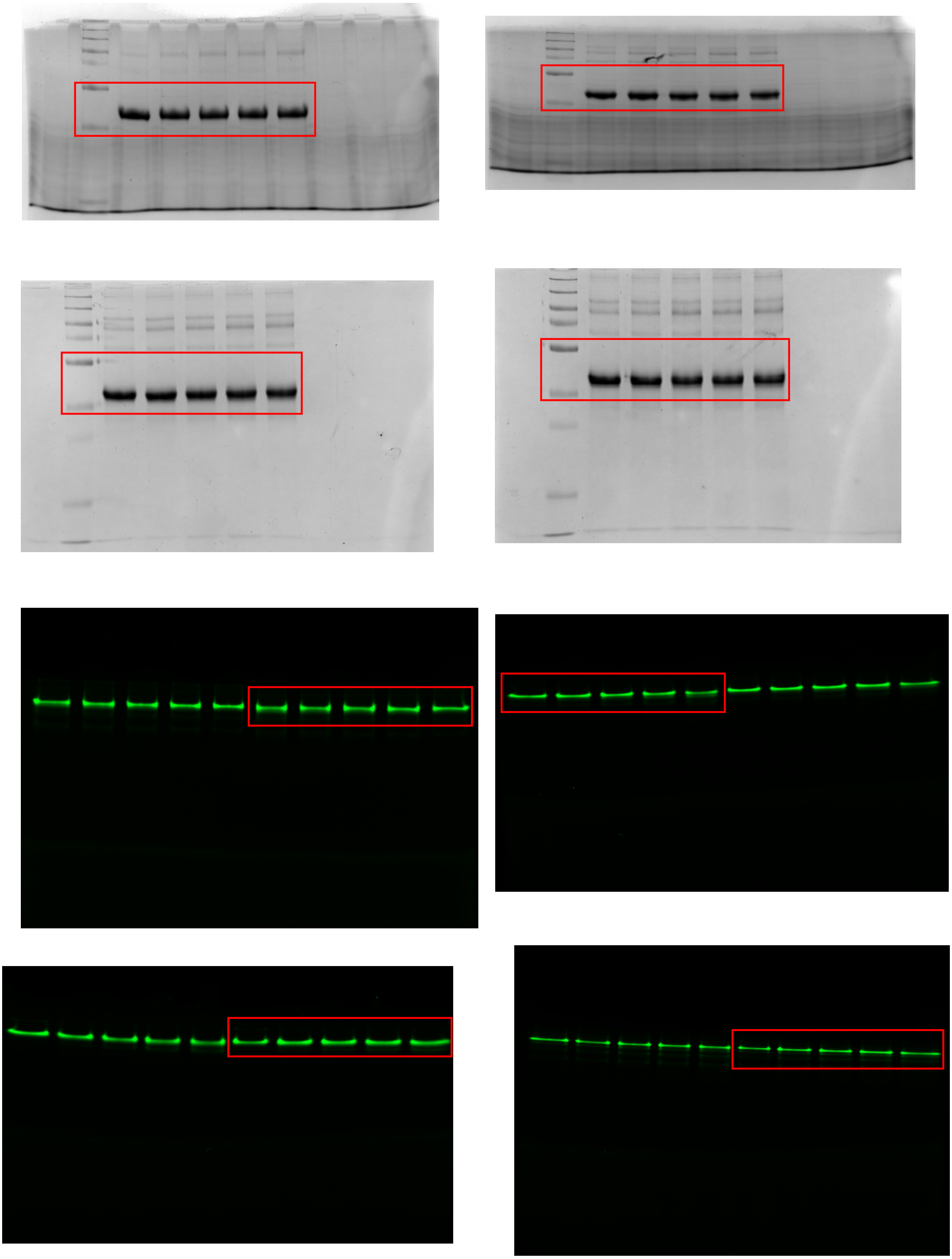

Original data for Figure 3

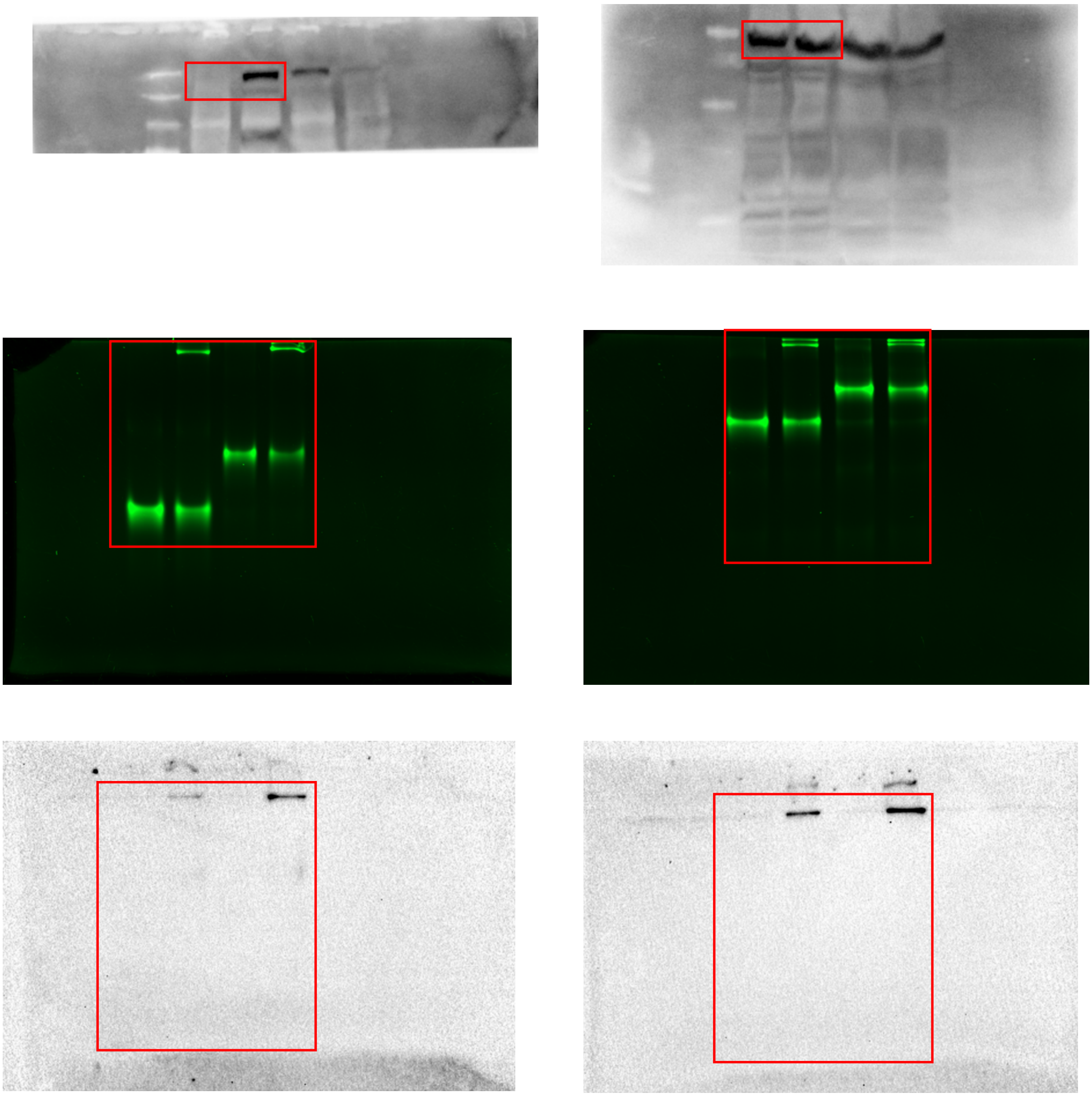

Original data for Figure 4

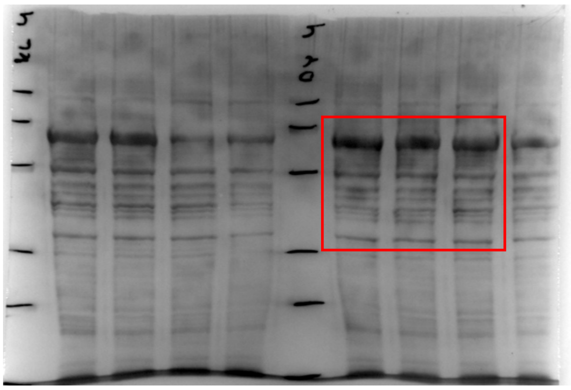

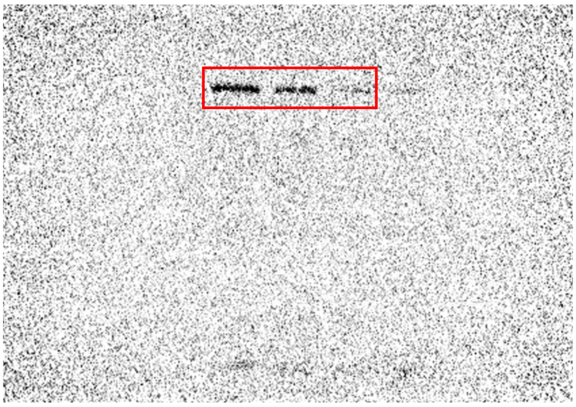

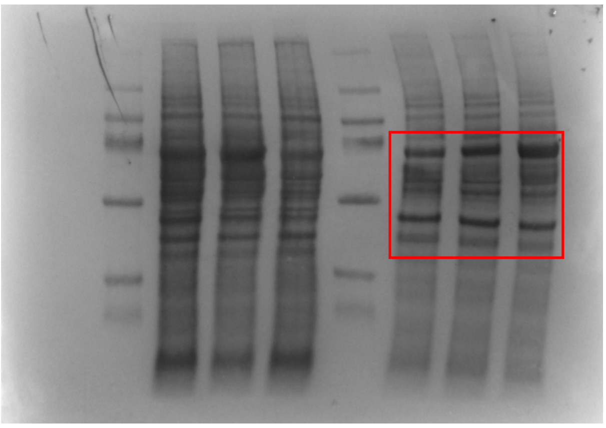

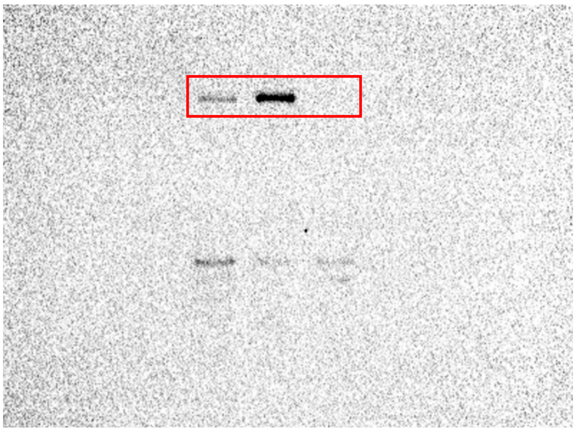

Original data for Figure 5

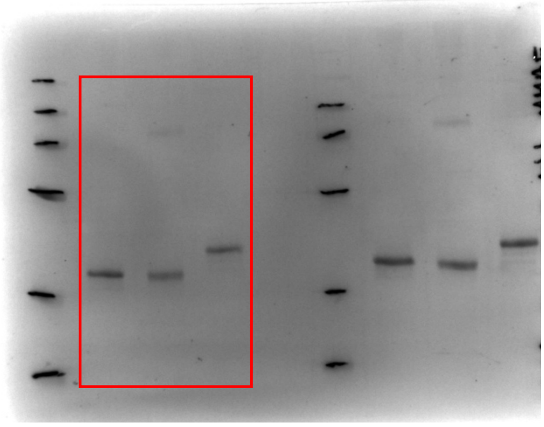

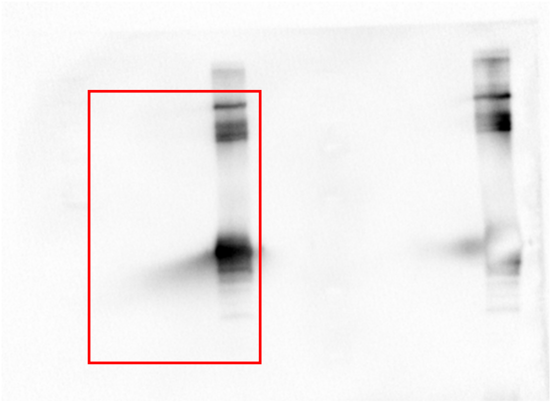

Supplement: Supplementary file 1 [file jm5c00782_si_001.pdf]
